# Structural Analysis of a Peptide Fragment of Transmembrane Transporter Protein Bilitranslocase

**DOI:** 10.1371/journal.pone.0038967

**Published:** 2012-06-20

**Authors:** Andrej Perdih, Amrita Roy Choudhury, Špela Župerl, Emilia Sikorska, Igor Zhukov, Tom Solmajer, Marjana Novič

**Affiliations:** 1 Laboratory of Chemometrics, National Institute of Chemistry, Ljubljana, Slovenia; 2 Faculty of Chemistry, University of Gdańsk, Gdańsk, Poland; 3 EN-FIST Center of Excellence, Ljubljana, Slovenia; 4 Institute of Biochemistry and Biophysics, Polish Academy of Sciences, Warsaw, Poland; University of Queensland, Australia

## Abstract

Using a combination of genomic and post-genomic approaches is rapidly altering the number of identified human influx carriers. A transmembrane protein bilitranslocase (TCDB 2.A.65) has long attracted attention because of its function as an organic anion carrier. It has also been identified as a potential membrane transporter for cellular uptake of several drugs and due to its implication in drug uptake, it is extremely important to advance the knowledge about its structure. However, at present, only the primary structure of bilitranslocase is known. In our work, transmembrane subunits of bilitranslocase were predicted by a previously developed chemometrics model and the stability of these polypeptide chains were studied by molecular dynamics (MD) simulation. Furthermore, sodium dodecyl sulfate (SDS) micelles were used as a model of cell membrane and herein we present a high-resolution 3D structure of an 18 amino acid residues long peptide corresponding to the third transmembrane part of bilitranslocase obtained by use of multidimensional NMR spectroscopy. It has been experimentally confirmed that one of the transmembrane segments of bilitranslocase has alpha helical structure with hydrophilic amino acid residues oriented towards one side, thus capable of forming a channel in the membrane.

## Introduction

Bilitranslocase (BTL) is a plasma membrane protein functioning as an organic anion carrier. It is found in liver cell membranes being involved in the uptake of bilirubin from blood to liver cells [1]–[6]. BTL is also expressed in other tissues including the vascular endothelium [7]–[9] or epithelia of the gastric mucosa [10]. It has been shown that BTL has an active role in the transport of many organic anions through the cell membrane [7], [11]–[13]. Therefore, it is also likely to be involved in the drug uptake, since carrier-mediated and active uptake of pharmaceutical drugs may be more common than is usually assumed, and should be considered as an essential step in rational drug discovery and development as reviewed in a recent perspective by Dobson and Kell [14]. Thus, it is of significant importance for the drug discovery process to understand at a mechanistic level the specificities of a known drug transporter for both drugs already in clinical use and potential drug candidates in development. An atomic resolution protein structure is needed for any detailed study of the drug-protein interactions and consequently for illuminating the mechanism of transport. Unfortunately, very few transmembrane proteins have their 3D structure solved using X-ray crystallography or NMR methods; less than 2% of solved structures in the PDB database can be ascribed to membrane proteins [15], [16]. The main experimental obstacle is low ability of membrane proteins to form a crystal structure, and even when soluble their inability of isotropic reorientation might prevent a suitable experimental approach using NMR spectroscopy [17]. Slow reorientation is the principal reason why it is difficult to obtain high resolution spectra of proteins incorporated in micelles or small bicelles. For this reason, it is crucial to select a proper solution medium for NMR studies of membrane proteins. Choice of detergent is empirical and protein-specific, and has to be optimized during the sample preparation procedure [18]. The solid state NMR technique is suitable to proteins of higher molecular weight, because in contrast to the solution state, the coherence lifetimes in the solid state are not affected by molecular tumbling [19].

Disappointingly, BTL is very problematic for experimental determination of its 3D structure although its primary structure has been available for some time [20]. BTL (UniProt O88750) consists of 340 amino acids with presumably four transmembrane regions which have not, however, been absolutely confirmed by neither experimental nor computational methods [21]–[23]. The amino-acid sequence of BTL displays no homology with known proteins, which makes it difficult to use a standard homology modeling approach in case of proteins with an unknown 3D structure. It is also not clear whether the BTL is present in the membrane as a monomer, or whether two or even three units should be associated for enabling active transport across the cell membrane [24].

**Figure 1:**
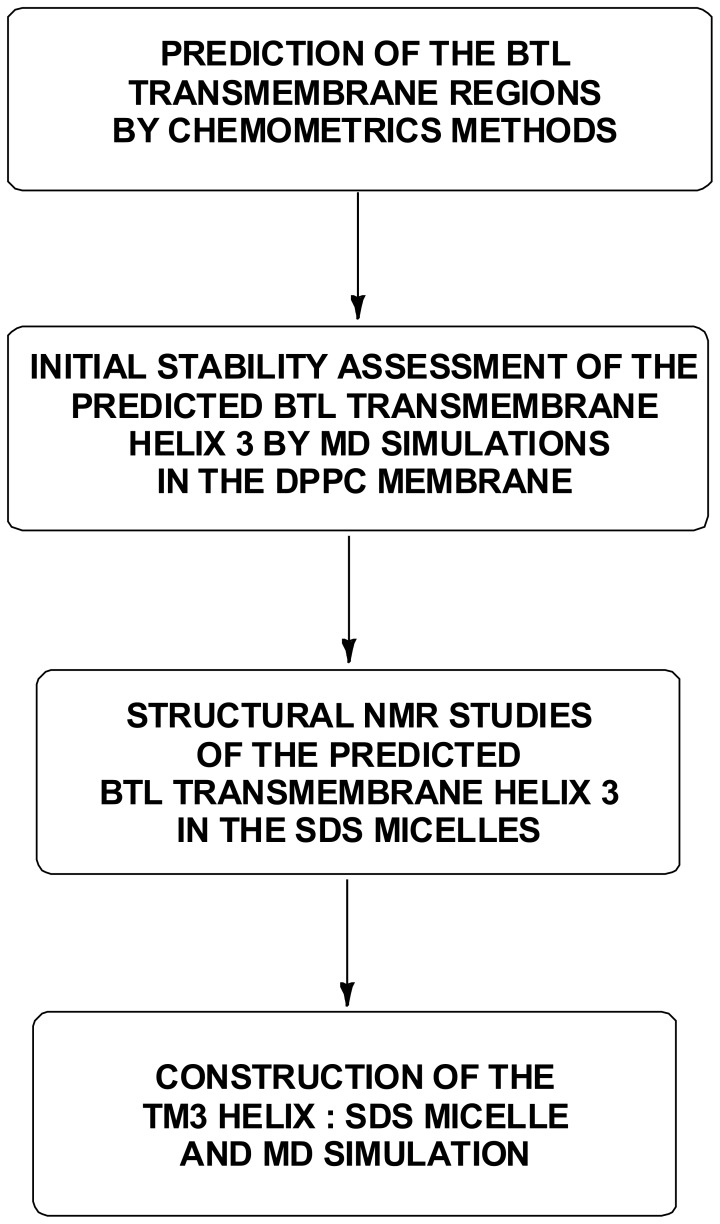
Schematic representation of the computational and experimental investigations of the BTL transmembrane region.

Having in mind the considerable interest for resolving the 3D structure of BTL on one side and all difficulties regarding parsimonious experimental data on the other, we have employed the chemometrics approach to predict the four alpha helical transmembrane subunits of BTL, which is in agreement with sparse available experimental data based on affinity-purified anti-sequence antibodies [22]. Molecular dynamics (MD) studies are used successfully to gain insight into the protein folding problem, biological function of the protein structure and in studies of ligand-protein interaction [25]–[29]. In the present work the initially predicted transmembrane regions of BTL have been reexamined and we substantiated that 3D structure of one of the transmembrane peptides (TM 3) is alpha helical by MD simulations in the standard DPPC (dipalmitoyl phosphatidyl choline) membrane. This result was further confirmed by the means of NMR spectroscopic study performed in an SDS (sodium dodecyl sulfate) micelle environment as well. The schematic workflow is presented in Figure 1.

It is to be stressed that BTL has no homolog in the PDB [20], and therefore, the conventional theoretical approaches for 3D structure prediction are not feasible in this case. Here we show for the first time 3D structure of one of the alpha helices of BTL spanning the membrane, which has been predicted by computational methods and confirmed experimentally. The other three predicted transmembrane segments of BTL are also in alpha helical conformation during molecular dynamics simulations; and in our subsequent work, we intend to examine the implications of the already resolved transmembrane structure for interactions with other helical peptides of the BTL protein sequence forming the channel and its transport properties for the uptake of drugs.

## Results and Discussion

### Prediction of BTL Transmembrane Regions

The transmembrane region prediction model [23] requires dividing the protein sequence into 20 residues long overlapping segments, each of which is then given to the model for prediction. Segmenting a 340 residues long BTL sequence yielded 329 such segments, which were then predicted by the model as either transmembrane or non-transmembrane. Only the long stretches of 10 or more consecutive overlapping segments predicted as transmembrane are considered for the final transmembrane region predictions. In the case of BTL, four such stretches of more than 10 consecutive overlapping segments were predicted as transmembrane, which were then further analyzed. These segments span over the residues 16–53 (19 segments), 65–103 (20 segments), 213–246 (15 segments), and 250–285 (17 segments). However, the whole transmembrane stretch is not reported as a final prediction because the terminal residues overlap with segments predicted as non-transmembrane. Hence, only the central residues of these four overlapping transmembrane segment stretches that are common to more segments predicted as transmembrane are considered and reported as the predicted transmembrane residues (see Text S1). Thus, the transmembrane regions of BTL, TM 1, TM 2, TM 3, and TM 4, were accordingly predicted to be at residues 24–45, 73–95, 221–238, 258–277 [23].

**Figure 2:**
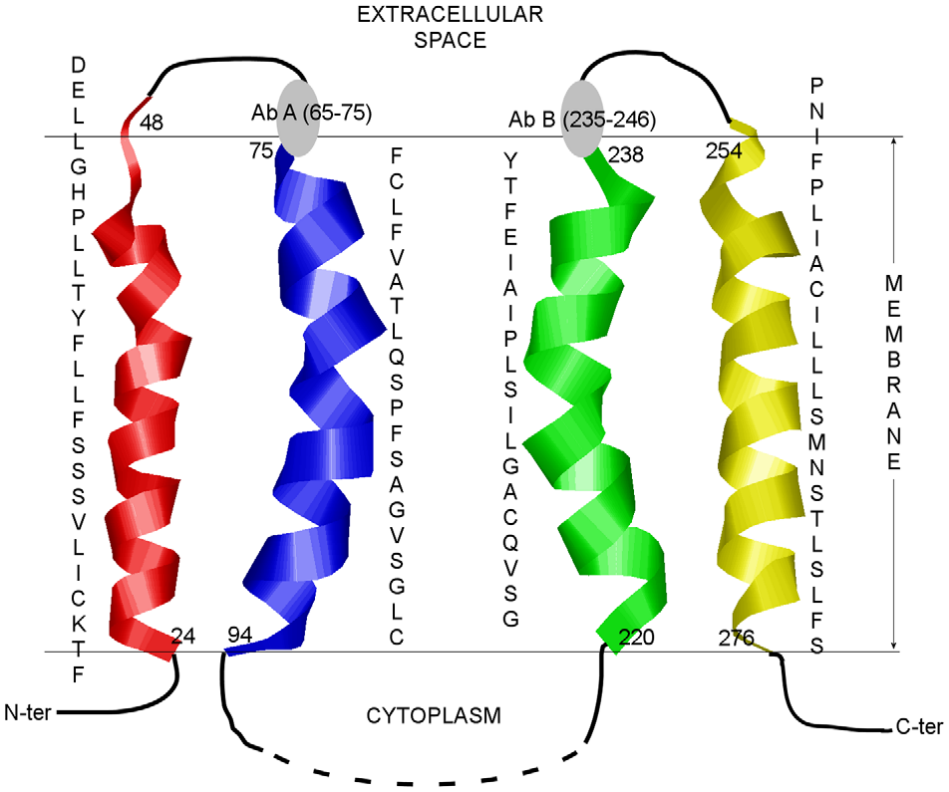
The four predicted transmembrane regions of BTL. The gray circles denote the antibodies tested.

The final stage of transmembrane region prediction includes statistical data obtained from position specific amino acid preference analysis. This was done to fine-tune the transmembrane boundaries. Instead of reporting only the central residues of the overlapping transmembrane segments as final predictions, we considered the residues that are more statistically favored. The terminal residues of all the segments in the transmembrane stretches predicted by the model were considered for this purpose, and were scored based on the statistically generated amino acid preference patterns (see Text S2). The position-specific scoring matrix has been already successfully applied in an automated multiple protein sequence alignment to classify proteins to a predefined family, in order to identify related proteins, which was reported in a survey of integral alpha helical membrane proteins [30]. In our research the best scoring terminal residues were reported as a final prediction if the region bounded by them met the minimum length criteria. In the Table S1, such combinations of terminal residues are listed and scored for the third stretch of transmembrane segments 213–246. The terminals of the segment 220–238 show the highest positive score of 13. Therefore, instead of the segment 221–238, the segment 220–238 with more statistically plausible terminals was now reported as the third transmembrane region of BTL. The other three transmembrane regions were predicted accordingly. Finally, the statistically improved four transmembrane regions of BTL, TM 1A, TM 2A, TM 3A, and TM 4A, were predicted to be at residues 24–48, 75–94, 220–238, and 254–276 respectively (Figure 2). It must be noted that the statistical scoring method was introduced to fine-tune the transmembrane region terminals, and therefore, the central parts of the predicted transmembrane regions remain the same in both the initial and final predictions with only difference at the terminal residues. A comparison of the predicted transmembrane regions with the results from other available predictors was also performed (see Text S3) [23]. Except for three predictors, the other tested predictors failed to predict all of the four proposed transmembrane regions of BTL. In our subsequent molecular dynamics simulations, we have checked the stability of both variants - TM 3 and TM 3A.

The four transmembrane regions predicted by the chemometric model are in accordance with experimental results from anti-sequence antibody studies that indicate the loops at N-terminus of TM 2 and C-terminus of TM 3 to be extracellular. Antibody A against the residues 65–75, the bilirubin-binding motif, establishes the segment to be extracellular. The bound antibody also inhibits transport function, confirming this sequence motif to be important for substrate binding and transport [6], [22]. Immediately next to it is the second proposed transmembrane region TM 2 (75–94) with two of its initial residues overlapping with the two terminal residues of the binding motif. Antibody B against segment 235–246 also shows the segment to be an extracellular one. Further, transport inhibition experiments signify the importance of the segment in transport mechanism [6], [24]. The segment B is located immediately after the third transmembrane region TM 3 (220–238) such that a few of the residues overlap. The observations from prediction model and antibody studies, therefore, indicate that the transporting channel possibly consists of the second (75–94) and third (220–238) transmembrane regions. The extracellular segments immediate to these two transmembrane regions assist in binding and guiding the ligands to the transporting channel. It was also concluded that both the N- and C-terminals of the protein are intracellular [23]. Owing to the functional importance of the transmembrane regions TM 2 and TM 3, a detailed study of these two transmembrane regions is crucial to understand the structural and functional mechanisms of the transporter BTL.

### Initial Stability Assessment of the Predicted BTL Transmembrane Helix 3 by the MD Simulation in the DPPC Membrane

**Figure 3:**
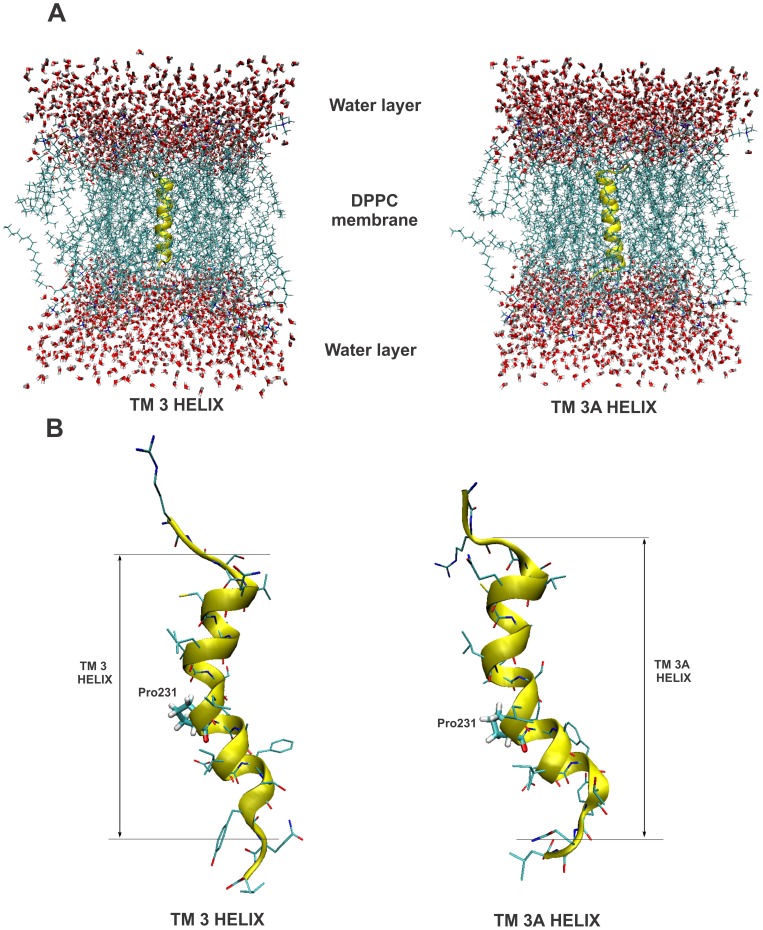
The initial configuration (A) and representative snapshots (B) of the alpha-helices TM 3 and TM 3A. In Fig. 3A the initial configuration of the alpha-helices TM 3 and TM 3A are inserted into the DPPC membrane. The helices are surrounded above and below by a layer of water molecules. In Fig.3B representative snapshots of the alpha helices TM 3 and TM 3A are from the MD simulation. The approximate borders of the alpha helical structures residue are also depicted. The BTL Pro231 residue, where the alpha helix kink is located is highlighted with a line model.

MD simulation was performed for the BTL transmembrane helix 3 (TM 3 helix residues 221–238) and its variant (TM 3A helix residues 220–238) to provide an assessment to see if the BTL sequences could adopt a stable helical conformation. The structure of the starting transmembrane helices TM 3 and TM 3A are shown in Figure 3.

The only difference between the two systems is that the TM 3A has an additional BTL amino acid Gly220 constrained to the alpha helical conformation as predicted by the model. The generated alpha helices were subsequently inserted into the membrane. The two additional amino acids that were added on the C-terminal and the N-terminal end of each of the alpha helices served to soften the boundary and enabled both ends of the alpha helix to explore more conformational space. The water molecules located on top and below the lipid bilayer mimicked the extracellular and intracellular compartments.

After performing the equilibration, the production MD simulations were yielding MD trajectories of the 20 ns length for each system. The produced 20 ns trajectories were first visually inspected for the overall conformational changes from the initial alpha helix structure, and it was revealed that in both systems the alpha helical conformation was retained. High resolution animations for TM 3A system is available in the Movie S1.

Representative snapshots of the alpha helices from the molecular dynamics (MD) simulation in the lipid membrane are presented in Figure 3. Secondary structure was predicted using the STRIDE program available in VMD software suite [31]. The conformational behavior in time showed comparable overall structure (see animation available in the Movie S1). Interestingly, the additional two residues located above and below the predicted alpha helical sequences did not adopt the alpha helical conformation; whereas the both predicted sequences TM 3 and TM 3A displayed highly conserved alpha helical conformation. The presence of proline in the predicted transmembrane sequence (Pro231) resulted in a kink formation in both helices due to the sterical interference with the backbone of the preceding turn inside a helix. This induced a bend of about 20–30° in both TM 3 and TM 3A helices. These observations are in accordance with the literature data and both helices remained stable in these conformations throughout the 20ns MD simulation [32]. Structural behavior of TM 3A was comparable with the one amino acid shorter TM 3 helix, and thus from the qualitative perspective an additional Gly220 provides also a stable helix in BTL structure within the performed MD simulation times.

In order to provide initial assessment of the alpha helix stability during the production stage, RMSD values for all backbone atoms initially generated in the alpha helical positions were calculated. Despite reasonably long 20 ns MD simulation runs a caveat must be stated that this simulation time still does not encompass sufficient conformational space necessary for a complete quantitative stability assessment of the helix under study. As during the MD simulation each alpha helix is allowed to move freely within the membrane, the occurring translational motion of the structure would obstruct the interpretation of the RMSD analysis. Thus, the RMSD values were calculated by aligning all conformations of the individual alpha helix to the last MD-generated alpha helix conformation. For the structural alignment, the backbone atoms (C', CA, N) were used for those residues that were chemometrically predicted to form the alpha helix. In addition, the average RMSD values along with standard deviations were calculated for these atoms using the average structure calculated from all the align frames in the MD trajectory (20000 structures) as a reference.

**Figure 4:**
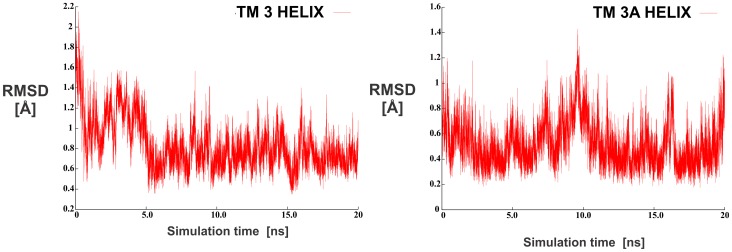
RMSD graphs of the backbone atoms for the alpha-helices TM 3 and TM 3A.

Calculated RMSD parameters indicate that alpha helix conformations of both TM 3 and TM 3A do not change extensively during the MD simulations, thus retaining the conformations obtained after the equilibration procedure. The average RMSD for helix TM 3 was 0.83 Å with a standard deviation of 0.24 Å (54 aligned atoms), and for the helix TM 3A the average RMSD value was 0.52 Å with the standard deviation of 0.21 Å (57 aligned atoms). We can conclude that a rather uniform and stable RMSD deviation was observed for both cases. To further analyze the conformation movement of the studied systems, RMSD graphs were plotted for both MD trajectories as a function of the simulation time. They are schematically presented in Figure 4 and display a high level of structural integrity during the simulation procedure.

**Figure 5:**
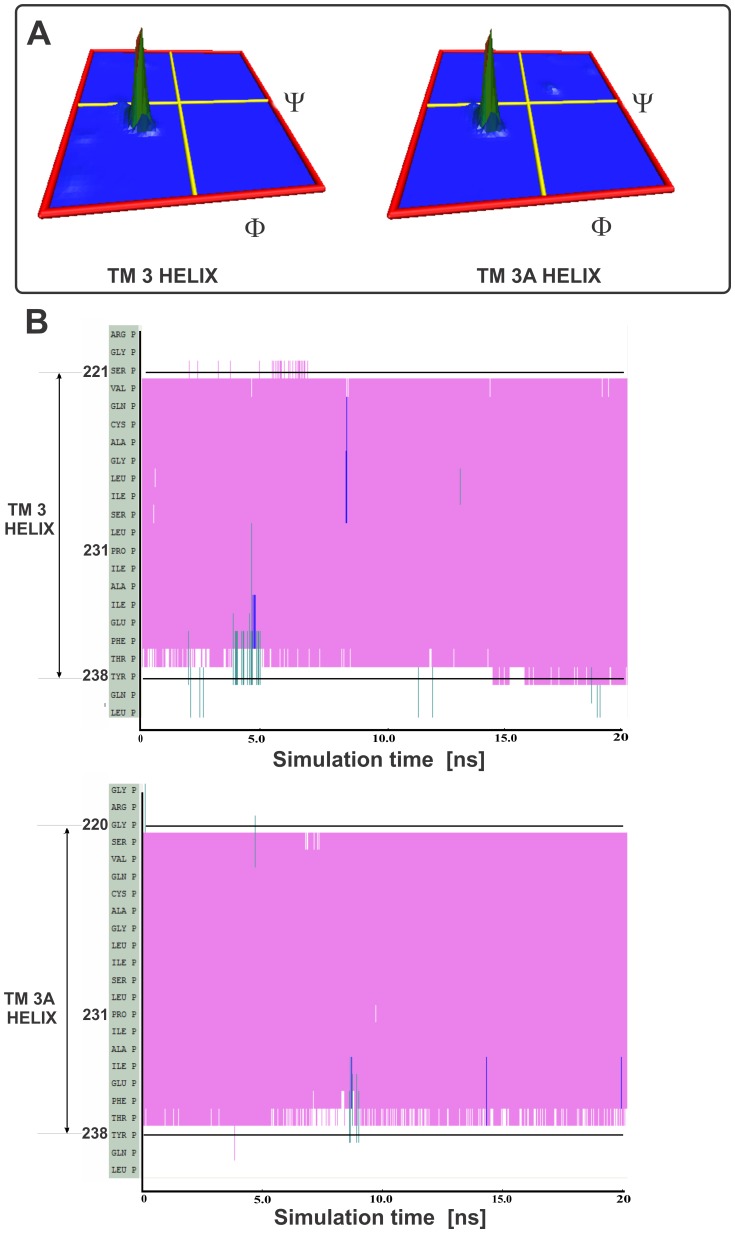
3D Ramachandran histograms for the backbone torsion angles φ and ψ (A) andtwo dimensional plots of the secondary structure analysis (B). In Fig 5A the analysis was performed for the residues that were predicted to from the transmembrane helix: 221–238 for the TM 3 helix and 220–238 for the TM 3A helix. Each exported conformation of the peptide in Fig 5B, generated by the 20 ns MD simulation was analyzed for is the secondary structure. Purple colour depicts the alpha helix structure, green indicates the turn structure and blue depicts the 3–10 helical structure. Selected residue numbers on the y-axis corresponds to the residues numbers of the BTL sequence.

The analysis of the MD trajectories was further focused on the distribution of the backbone torsion angles φ and ψ for both systems during the MD simulations using the available graphical tools. Whereas the standard Ramachandran plots can efficiently display the φ and ψ backbone torsion angles for each residue in a protein for a single frame only, further extension to three dimensional histograms is useful for viewing the frequency distribution of all dihedral backbone angle values observed during the MD simulation. As only certain values for these angles are allowed in the alpha helical conformation, this provides a way of assessing the integrity of the alpha helical conformation in both simulated systems. The obtained 3D Ramachandran histograms are displayed in the Figure 5B for the backbone angles of those residues that were predicted to be in the alpha helical conformation. The initial φ and ψ torsion angles of −57° and −47°, which were used to constrain the predicted BTL protein sequence to alpha helical conformation, were in line with the experimental observations. In alpha helical conformation the ψ dihedral angle of one residue and the φ dihedral angle of the subsequent residue sum to approximately −105° and that residues in α-helices typically adopt backbone φ and ψ dihedral angles around −60° and −45° respectively [32]. As shown in Figure 5A, a very uniform and stable distribution of backbone dihedral angles reveal the values of torsion angles typical for the alpha helix structures. Thus, residues that were modeled into the alpha helical conformation retained such conformation throughout of the molecular dynamics simulation of the protein inserted in the DPPC membrane.

The VMD program was used to produce two dimensional plots where the simulation time from 20 ns molecular dynamics trajectory is plotted against the secondary structure analysis of each frame of the protein structure. The overall plots for both helices are presented in Figure 5B. Apart from the occasional drift of the final residue in the helix (Tyr238) the secondary alpha helix structure seems to be fully stable.

The overall conclusion of our initial MD assessment was that BTL sequences TM 3 and TM 3A predicted by the chemometrics approach to encompass the third transmembrane region of the BTL can adopt a stable alpha helical conformation when inserted into the DPPC membrane during 20 ns MD simulation. The inclusion of Gly220 in TM 3A also enabled the stable conformation of the prolonged alpha helix with virtually all qualitative and quantitative characteristics preserved (RMSD, Ramachandran histogram, secondary structure analysis) compared to the one amino acid shorter TM 3 helix. A subsequent experimental investigation was performed to gain an even more detailed and precise structural insight.

### NMR Study of TM 3

**Figure 6:**
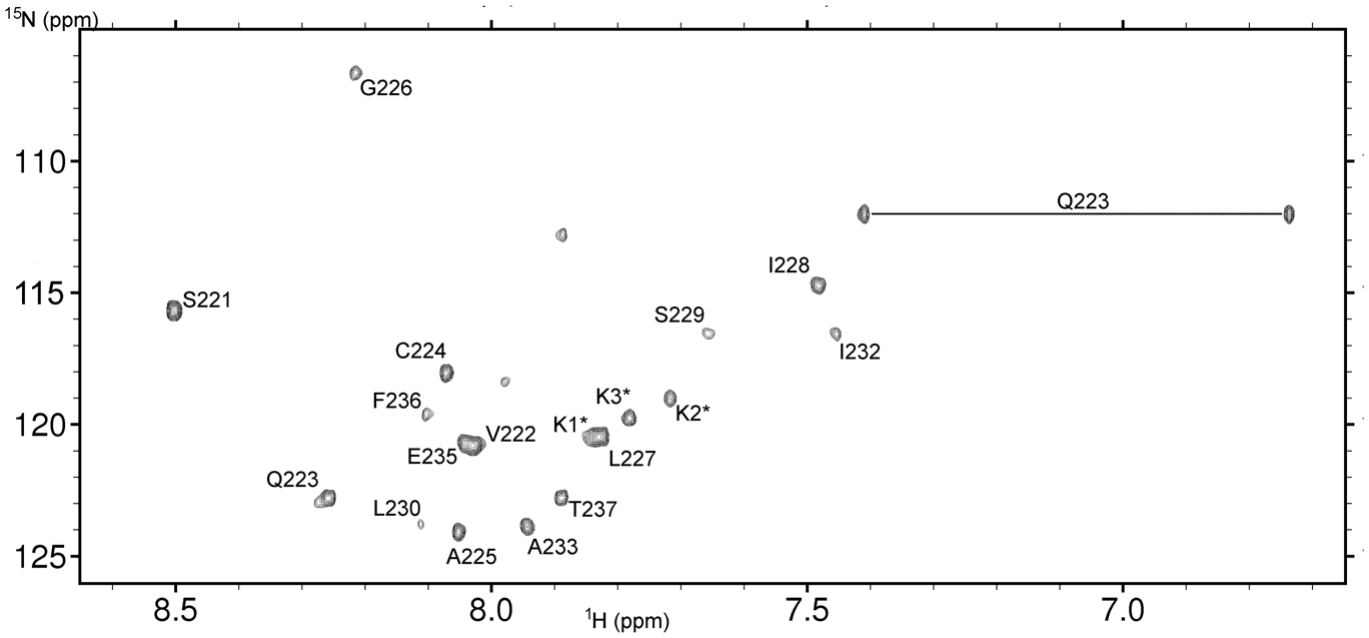
2D ^1^H-^15^N HSQC spectrum. It is acquired on Varian VNMRS 800 NMR spectrometer at 298 K on natural abundance of ^15^N isotope. The sequence-specific assignments of main conformation of GSVQCAGLISLPIAIEFTKKKK peptide are presented. The resonance signals coming from side chain NH_2_ group for Gln4 are also shown.

Inspection of 2D homonuclear NMR spectra indicates that after the addition of SDS-d_25_ to solution to increase solubility, the peptide reveals a well defined 3D structure. Assignments of ^1^H resonances were achieved with standard procedure on the base of 2D ^1^H-^1^H TOCSY acquired with mixing times 80 ms and 15 ms, and 2D ^1^H-^1^H NOESY recorded with mixing time 120 ms data sets [33]. Collected homonuclear NMR spectra were supplemented by heteronuclear 2D ^1^H-^15^N and ^1^H-^13^C HSQC (Heteronuclear Single Quantum Coherence) experiments recorded on natural abundance of ^15^N and ^13^C isotopes (Figure 6). Finally, more than 87% of expected ^1^H, ^13^C, and ^15^N resonances in peptide were successfully assigned for the 22 residues long synthetic peptide that corresponds to 220–237 residues of the BTL sequence extended by four lysines. Details on the synthetic peptide are given in the section Materials and Methods.

The chemical shift of ^13^C^β^ resonance (27.83 ppm) in Cys224 confirms that the thiol group exists in a reduced state [34]. Due to strong T_1_ noise coming from water resonance on heteronuclear ^1^H-^13^C HSQC we could not assign ^13^C^β^/^1^H^β^ and ^13^C^γ^/^1^H^γ^ correlations for Pro231 and a geometry of Leu230– Pro231 peptide bond was deduced from an existence of a cross peak between ^1^H^α^ Leu230 and ^1^H^δ^ Pro231 and established as *trans*. On the other hand, we have noted several unassigned cross peaks on 2D heteronuclear and homonuclear NMR spectra corresponding to the minor conformation of peptide, which is probably formed due to *cis*−/*trans*- isomerization of peptide bond between Leu230-Pro231.

### Conformational Analysis of the Peptide 3D Structure in SDS Micelle

**Figure 7:**
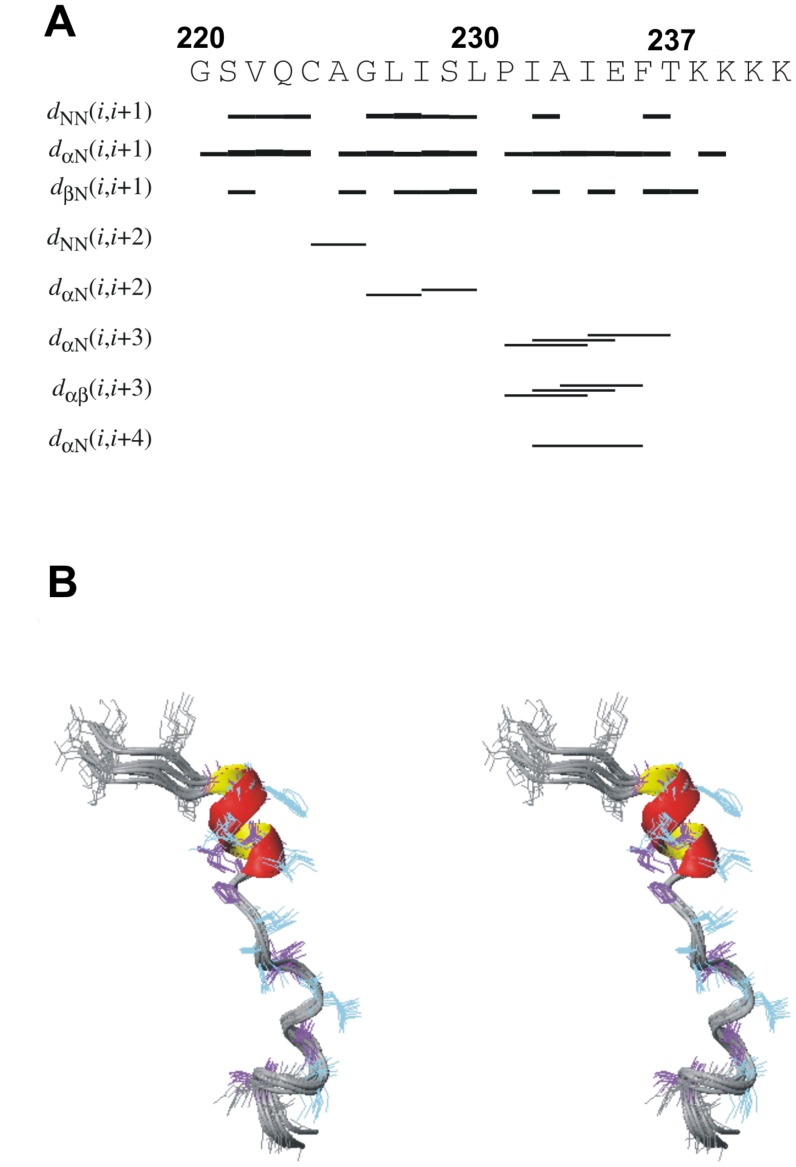
Sequence plot of NOESY distance constraints (A) and stereo-view of 10 conformers of studied peptide (B).

The 2D ^1^H-^1^H NOESY experiment acquired with the mixing time 120 ms displays several medium range *d*_HαHN_(*i,i+2*), *d*_HαHN_(*i,i+3*) distance contacts assigned to through space interactions between Ile232 ^1^H^α^ – Glu235 ^1^H^N^, Ile232 ^1^H^α^ – Phe236 ^1^H^N^, Ala233 ^1^H^α^ – Ile234 ^1^H^β*^ protons (Figure 7A) which are characteristic for α-helices [33]. The chemical shifts analysis performed with program TALOS+ [35] also suggests the helical conformation for Ile234 and Glu235 (see Figure S1).

Initial rounds of 3D structure evaluation performed with program CYANA [36] confirmed our experimental data exhibiting existence of the short α-helix in the region Ile232– Phe236. The α-helical fragment in the C-terminal part of the peptide is also preserved during the 5 ns time-averaged molecular dynamics in *an explicit* SDS micelle in the parm99 force field of AMBER 11.0 package. The backbone torsion angles for a couple of other residues (Val222, Gln223, Cys224 and Leu227) are located predominantly in a helical region on the Ramachandran plot. A stereo-view of the ensemble of the last 10 structures of peptide obtained with the MD simulations with time-averaged distance restraints (TAV) is demonstrated in Figure 7B. The peptide has a helical conformation with RMSD for backbone atoms 0.39±0.15 Å for the fragment Ala225– Thr237. All experimental constraints used in structure calculations and parameters demonstrated quality of the final 3D structure are presented in Table S2.

**Figure 8:**
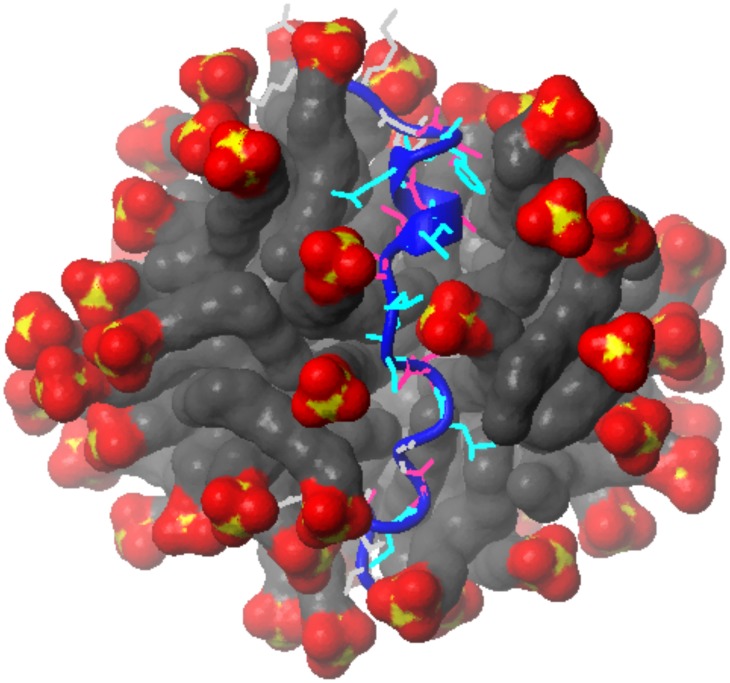
High-resolution 3D structure of peptide in SDS micelle. The side chain of residues in the hydrophobic core (Val222, Ala225, Ile228, Pro231, Ala233, Ile234 and Thr237) and those exposed to water (Cys224, Ser229, Leu230, Ile232, Glu235 and Phe236) are shown in violet and cyan, respectively.

**Figure 9:**
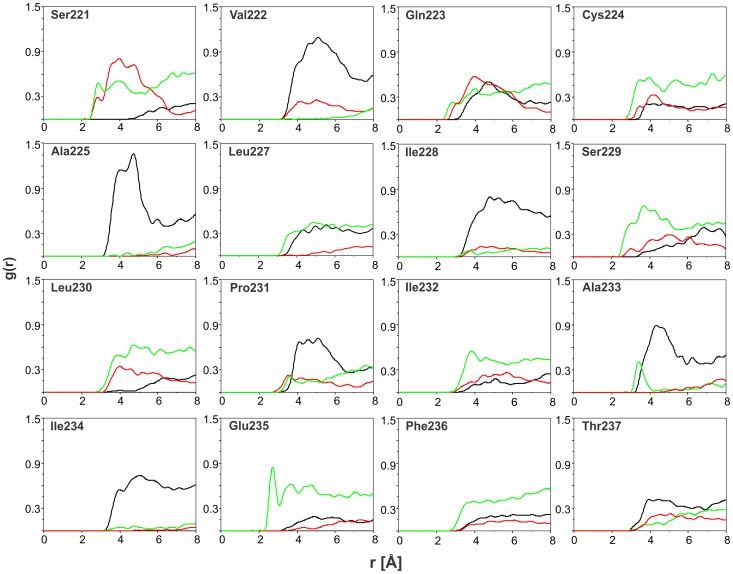
Radial distribution function (RDF) plots. RDF of hydrophobic (black), hydrophilic (red) parts of SDS micelle and water molecules (green) and the heavy atoms in side chains for all residues with the exception of Gly220 and Gly226.

During molecular dynamic simulations, the peptide diffused from the hydrophobic core of the SDS micelle to the interface to adopt more favorable conformation from the energetic point of view. After approximately 5 ns of molecular dynamic simulations the position of the peptide was fixed and it remained bound to the SDS surface (Figure 8). The peptide – micelle and peptide – water interactions were analyzed by the radial distribution function (RDF) obtained with the program Ptraj from AMBER 11.0 program suite (Figure 9). Inspection of the RDF data demonstrated that several residues have side-chains exposed to water. These are Cys224, Ser229, Leu230, Ile232, Glu235 and Phe236. The side-chains from Val222, Ala225, Ile228, Pro231, Ala233, Ile234 and Thr237 are rather buried in hydrophobic part of SDS micelle (Figure 9).

### The Membrane Part of Peptide Constitute Ala225-Thr237 Region

3D structures obtained in MD simulations with AMBER show that three residues on the N-termini did not reveal any fold in water/micelle solution (Figure 7B). Detail inspection of presented RDF data shows that Ala225 is probably the first residue from the N-termini with side chain buried in hydrophobic core of SDS membrane (Figure 9). On the other side, Thr237 probably is the last residue in hydrophobic surrounding. This make us able to define the Ala225– Thr237 part of studied peptide as a region intensively interacted with SDS micelle. The calculation distances between C^α^ atoms in Ala225 and Thr237 demonstrated that it is oscillating around 22 Å during last 200 ps of molecular dynamic simulations (Figure S2) which is in a good agreement with the thickness of the cell membrane (∼ 20 Å).

### Positioning Peptide in SDS Micelle

**Figure 10:**
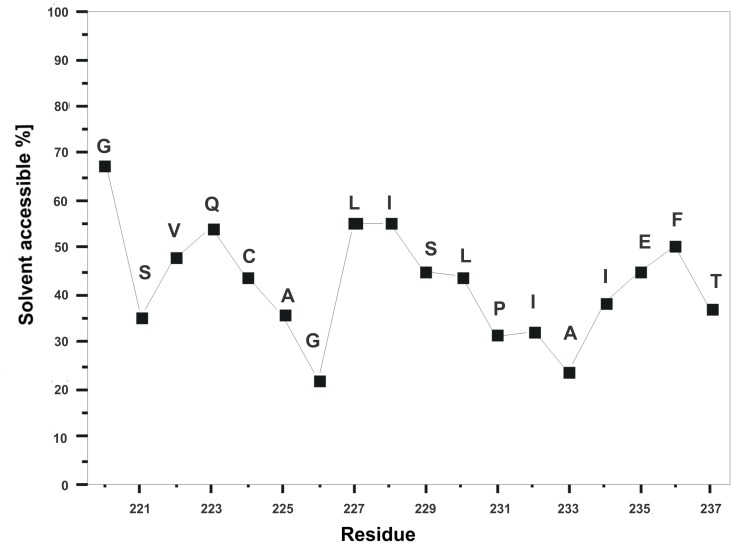
The average surface accessibility of amino acid residues. 10 conformations obtained during 5 ns of the MD simulations of the peptide were considered.

Detail analysis of the RDF data obtained with Ptraj program in AMBER 11 software suite reveals some conclusions about the positioning of the peptide in the SDS micelle. In particular, there are several residues (Ala225, Ile228, Pro231, Ala233, and Ile234) with the side-chains buried into the hydrophobic part of a micelle (Figure 9). Taking into account the position in the sequence, these residues more or less correspond to helix turns and are located on one side of the helix. The other side of the helix, composed by Ser229, Leu230, Ile232, Glu235, and Phe236, is more hydrophilic and the side-chains are exposed to aqueous phase, which is confirmed by the water-accessible surface calculated with MOLMOL [37] (Figure 10). Presented data have a sinusoidal form which is typical for a helix structure. For instance, the Pro231– Ala233 part of the peptide sequence, which reveals a relatively small surface accessible to water, constitutes part of the helix buried inside the hydrophobic part of an SDS micelle. The next three residues (Ile234– Phe236) exhibit an increase of accessible surface exposed to solution. Data obtained for the Thr237 show that this last residue is more buried in hydrophobic part of SDS micelle.

### In Summary, Towards Revealing the Potential Mechanism of Transport of BTL

Our present study started with the prediction of four transmembrane segments of BTL using the developed chemometrics model. The predictions were fine-tuned using statistical data. The proposed transmembrane regions are also in accordance with the results from previously performed antibody studies [22], [24]. For all predicted segments, we have observed the stability of their 3D structure by MD simulation in a model membrane environment. Both TM 3 and TM 3A were shown to retain stable alpha helical conformation in MD. The experimental NMR results in SDS micelle have also confirmed the alpha-helix structure of one of the helices, TM 3A, the final statistical variant of the third transmembrane region. Furthermore, the orientation of hydrophilic/hydrophobic amino acids in the 18-residues long transmembrane segment TM 3A and the analysis of their radial distribution functions support the hypothesis that it could form a building block of a hydrophilic channel within the membrane. This is in agreement with antibody studies, which along with TM 3, also strongly suggest TM 2 to be an important functional unit in the transport channel formation. The role of TM 1 and TM 4, whether they form the channel functional for transport of hydrophilic molecules, is not yet known. While peptide TM 3 consists mainly of amino acids that are weak or strong electron donors, TM 2 also contains those that are electron acceptors. Both TM 2 and TM 3, however, have aromatic amino acids – phenylalanine and tyrosine - that are weak electron acceptors at their extracellular terminal overlapping with the binding motifs. NMR studies revealed Glu235 of TM 3 to be exposed to the aqueous phase and thus forming the transport channel wall. Glutamic acid is a strong electron donor; and is also forming the substrate-binding region (corresponding to Ab B, residues 235–246) [22], [24]. It is therefore probable that Glu235 plays an important role in substrate binding and transportation through a BTL channel acting as a hydrogen bond acceptor for the substrate molecules. Another amino acid of TM 3 that may potentially play a role in transport mechanism is the nucleophilic Ser229, which is also exposed to the hydrophilic environment. In partial support of this hypothesis are the results of several previous experimental studies [38], in which the transport of a number of flavonoids as well as nucleotides and nucleosides by BTL were evaluated. The structure - activity correlations of molecules in these series strongly associated the hydrogen bond formation parameters with their experimental transport properties in BTL [11], [13]. In our subsequent studies, we plan to analyze the peptide TM 2 and the transport mechanism in detail. We would also like to analyze the inter-relation of the four transmembrane peptides of BTL to get a more detailed picture regarding the structure and functional mechanism of the protein.

## Materials and Methods

### Initial Prediction of Transmembrane Regions of BTL

The transmembrane regions of BTL were initially predicted using the first version of the alpha transmembrane region prediction tool [23]. It is a data-driven model based on mathematical descriptors of the protein segments derived from an amino acid adjacency matrix. The model is built using a counter-propagation neural network (CPNN), a non-linear supervised learning method. The model shows an overall prediction accuracy of 90.75% when tested with an external validation set. Details are given in Text S4.

The developed model predicts the transmembrane regions of BTL from its sequence information alone. The 340 residues sequence was divided into overlapping segments of 20 amino acids each by shifting one residue at a time, therefore yielding 329 such segments. The model then predicted for each segment whether it is transmembrane or not.

### Final Transmembrane Region Prediction of BTL with Incorporation of Statistics

The second version of the alpha transmembrane region prediction model incorporates amino acid preference data for final prediction. It is used to score the probable transmembrane region terminals (see Text S2). We considered all possible combinations of terminal residues from the initial overlapping segments of BTL predicted as transmembrane. This generated a list of probable transmembrane segment terminals for each region. One of the top 3 scoring segments that meet the length and position criteria is reported as a transmembrane region in final prediction.

### Preparation of the Predicted BTL Transmembrane Helices TM 3 for the Molecular Dynamic Simulation

Two molecular systems were constructed using CHARMM molecular modeling environment [39]. The initial conformation for the amino acid sequence that was predicted to comprise the transmembrane region TM 3 (BTL residues: 221–238) SVQCAGLISLPIAIEFTY [23] from the initial chemometric prediction model and another conformation of the new chemometric prediction model with statistics TM 3A (BTL residues: 220–238) GSVQCAGLISLPIAIEFTY discussed in this article were generated by using CHARMM topology and structural libraries [40]–[43]. Subsequently, an alpha helix conformation for each linear sequence was generated by constraining the backbone torsion angles φ and ψ to the values of −57° and −47° for each amino acid backbone angle respectively [44]. When building the initial sequences, two additional amino acids corresponding to the residues located prior to the start (219–220 BTL residues RG for TM 3 system and 218–219 BTL residues GR from the TM 3A system) and subsequent to (239–240 BTL residues QL for both TM 3 and TM 3A systems) the end of the transmembrane helix on the BTL sequences were added on the C-terminal and N-terminal end of TM 3 and TM 3A. These additional amino acid residues were not constrained to the alpha helical conformation.

For the construction of the lipid-protein systems CHARMM-GUI Membrane builder generator was exploited [45]. The CHARMM generated alpha helices were first oriented along the principal Z-axis. DPPC lipid molecules were selected to represent the lipid bilayer [46]. A rectangular box consisting of two layers of DPPC lipids along with 12 Å thick layers of water molecules above and below the lipids surface was constructed. Based on the system size determined, CHARMM-GUI was used to construct the individual parts such the lipid bilayer around the protein and additional water molecules to fully solvate the system. Each alpha helix was inserted into the membrane using the insertion method, where a protein is inserted into a pre-equilibrated lipid bilayer [45]. Finally, all building parts were assembled together to form the molecular systems with the size of 18874 (TM 3 system) and 18130 atoms (TM 3A system), respectively.

### Molecular Dynamics Simulation Procedure

Molecular dynamics calculations were performed utilizing the CHARMM molecular modeling suite [39]. CHARMM parameter and topology files (version 27) for proteins and lipids were utilized to specify force field parameters describing the protein and the lipid DPPC molecules [40]–[43]. To equilibrate the membrane the following scheme was used resulting in a total of 375 ps simulation time [45]. The system was first minimized for 500 steps using the steepest descent method followed by 500 steps of the modified Adopted Basis Newton-Raphson method with the following set of force constants: K1 defined the force constant applied to the protein backbone, K2 force constant was applied to the amino acid side chains, Kwforce force constant was designed to keep the water molecules away from the hydrophobic core, Ktforce force constant constrained the lipid tail, and finally Kmforce force constant constrained the movement of the lipid head groups. The values of the used constants for each performed equilibration step are collected in Table 1 [45].

**Table 1:** Values of the force constants used during the equilibration MD steps of the TM 3 and TM 3A helices inserted into the DPPC membrane (values are in kcal/mol/A^2^) [45].

	Equlibration					
	step 1	step 2	step 3	step 4	step 5	step 6
**K1**	10	5.0	2.5	1.0	0.5	0.1
**K2**	5	2.5	1.0	0.5	0.1	0.0
**Kwforce**	2.5	2.5	1.0	0.5	0.1	0.0
**Ktforce**	2.5	2.5	1.0	0.5	0.1	0.0
**Kmforce**	2.5	2.5	1.0	0.5	0.1	0.0

In the first two equilibration steps (1–2) the system was simulated twice for 25 ps using the Langevin dynamics along with a 1 fs integration time step. Further four equilibration steps (3–6) were performed with the standard molecular dynamics using leapfrog integration scheme. To reduce the possible problem with the numerical integration with the uncorrelated system, 1 fs time-step was used only in the third step with the total equilibration time of 25 ps. In the next stages of the integration scheme, 2 fs step along with SHAKE algorithm was applied. The simulation times for steps 4–6 were 100 ps long. Production trajectories were generated using a leapfrog integration scheme and 2 fs simulation step using SHAKE algorithm. 20 ns long MD simulation was performed for both TM 3 and TM 3A trajectories. Results of the MD simulations were visualized and analyzed using VMD software [29], [47] and RMSD diagrams were created using Gnuplot program [48]. 20000 MD generated configurations for each simulated alpha helices were exported for further studies again using VMD available MD analysis tools.

### Synthetic Peptides – Commercial Source

Synthetic peptides used in this study were purchased from CASLO Laboratory, Denmark (www.caslo.com). The peptide (TM 3) is a lyophilized trifluoroacetate salt with four lysines at the C-terminus with the sequence GSVQCAGLISLPIAIEFTKKKK and purity 93.87%. Four lysines were added by the producer because of extreme hydrophobicity of the peptide and therefore problems with synthesis and purity.

### NMR Sample Preparation

The NMR sample was obtained by making a solution of about 1 mM peptide in partially deuterated water (90%/10% H_2_O/D_2_O) containing about 32 mg of deuterated sodium dodecyl sulfate micelles (SDS-d_25_) (Sigma Aldrich). The concentration of SDS-d_25_ exceeded the critical micelle concentration (8.3 mM) and SDS-d_25_ : peptide ratio was adjusted to approximately 40∶1 to be sure that the peptides were in micelle bound state. To increase homogeneity of the peptide:micelle complex, the NMR sample was placed into an ultrasound bath for nearly 20 minutes. The pH was measured just before the start of the NMR experiments and its uncorrected value was 6.5.

### NMR Measurements

All NMR data sets were acquired at 303 K on a Varian VNMRS 800 NMR spectrometer (^1^H resonance frequency 799.81 MHz) equipped with four channels, gradient unit along z-axis, DirectDrive console and ^1^H/^13^C/^15^N probe-head with inverse detection. Homonuclear 2D ^1^H-^1^H TOCSY spectra were recorded with mixing times 15 and 80 ms using MLEV-17 pulse scheme for spinlock (Bax & Davis, 1985). 2D ^1^H-^1^H NOESY data sets were acquired with 80, 120 and 150 ms mixing times for exclusion of the spin-diffusion effect. To get access to ^13^C and ^15^N chemical shifts the heteronuclear 2D ^1^H –^15^N and ^1^H –^13^C HSQC experiments were performed on natural abundance of ^13^C and ^15^N nuclei. All chemical shifts were referenced with respect to external 2,2-dimethyl-2-silapentanesulfonic acid (DSS) using Ξ = 0.251449530 and Ξ = 0.101329118 ratios for indirectly referenced ^13^C and ^15^N resonances, respectively [49]. All recorded NMR data were processed by NMRPipe software [50] and analyzed with the Sparky program [51].

### 3D Structure Calculation of Peptide

3D structure calculations were done using ^1^H-^1^H distance constraints evaluated on the base of 2D homonuclear NOESY spectrum acquired with mixing times 120 ms. The manually selected NOESY cross peaks yielded 180 nontrivial distance constraints (107 intraresidual, 58 sequential, and 15 medium range) which were applied for structure solution. The calculation protocol started with generating 200 initial structures created with randomly chosen torsion angles. The calibration of peaks’ volume to distance constraints was done with the CALIBA program in semiautomatic way. Finally, simulated annealing procedure with 10000 steps of molecular dynamic in torsion angle space was performed with the ANNEALING program. All programs are included in the CYANA 2.1 [36] software suite.

### Construction of Peptide: SDS Complex and Molecular Dynamic Simulations

Molecular dynamics simulations were carried out with the program AMBER 11 [52]. Micelle preparation was initiated by construction of a single molecule of sodium dodecyl sulfate (SDS) on the basis of previously published parameters [53], [54] using united atoms presentation and all bonds in *trans* configuration. According to previously established protocol [52], a starting model containing 60 monomers of SDS was subjected to minimization in vacuum. Later, minimized micelle was placed in a cubic box enlarged by about 8 Å in each direction that included water molecules. As a result, 7126 water molecules were added to simulated peptide:micelle complex increasing the total number of atoms up to 22458. Finally, a 1 ns simulation was carried out in order to reach an equilibrium density of the entire system. The final density was 1.0080 g/mL which is in agreement with reported experimental value of 1.0093 g/mL for SDS/water solution [55].

Molecular dynamic procedure was started by positioning the peptide into the simulation box with its center of mass coinciding with that of the micelle. The initial peptide structures were taken from results of calculations with the CYANA program. Owing to micelle spherical symmetry, orientation of the peptide was not important. Taking into account the pH of NMR sample, the side chains of four lysines and N-terminus were defined as protonated, whereas the side chains of one glutamate and C-terminus were negatively charged. The chloride ions were used to neutralize a total charge +3 of the entire system.

To remove the initial bad contact between the peptide and the micelle core and to prevent penetration of water during equilibration the peptide and bulk water were kept under weak harmonic constraints with force constants of 10 and 5 kcal/(mol×Å), respectively. Those constraints were removed after 20000 steps of minimization (the steepest descent method). Later, the entire system was minimized for 20000 steps without any constraints. Thereafter, the whole complex was subjected to molecular dynamic simulations under the constant pressure and the temperature of 301 K for 5 ns with TAV distance restraints derived from NMR spectroscopy. The interproton distances were introduced with the force constants f = 20 kcal/(mol×Å^2^). The geometry of the peptide groups was kept fixed according to NMR data (all *trans*) with the force constant f = 50 kcal/(mol×rad^2^). MD simulations were performed with a time step 2 fs and 9.0 Å cutoff radius. The coordinates were recorded at 4 ps (2000^th^ step) each and 10 conformations obtained in the last steps of MD simulation were chosen for final structure analysis.

Obtained 3D structures were analyzed with Ptraj program included in the AMBER 11.0 package. To characterize interactions of the peptide with the micelle or aqueous phase, the radial distribution functions for peptide side chains and negatively charged groups, hydrophobic part of the SDS-d_25_ micelle and water, were calculated. The presented data were evaluated on the average of over the last 200 ps of MD simulation.

## Supporting Information

Figure S1 **Prediction of conformation for Ile234 with the program TALOS+** [**35**] **based on reported chemical shifts.**
(DOC)

Figure S2 **Distance between C^α^ atoms in Ala225 and Thr237 calculated on base last 200 ps of trajectory in molecular dynamic simulations performed with the program AMBER 11.**
(DOC)

Table S1 **Scoring all the possible combinations of terminal residues for the third transmembrane stretch of BTL.**
(DOC)

Table S2 **Structural statistics of distance constraints used for high-resolution 3D structure calculations and quality ensemble of 10 NMR derived structures of 22 residues long peptide on the last steps of MD simulations with TAV.**
(DOC)

Text S1 **Prediction of BTL transmembrane regions.**
(DOC)

Text S2 **Statistical calculation of amino acid position preference.**
(DOC)

Text S3 **Comparison with other predictors.**
(DOC)

Text S4 **Details on Counter-propagation neural network model.**
(DOC)

Movie S1 **High resolution animations for the MD simulation for TM 3 molecular systems.**
(MPG)

Movie S2 **High resolution animations for the MD simulation for TM 3A molecular systems.**
(MPG)
