# Supplementary material for: Structural Analysis of a Peptide Fragment of Transmembrane Transporter Protein Bilitranslocase
Source: PLoS One. 2012 Jun 20;7(6):e38967. doi: 10.1371/journal.pone.0038967 (PMC3380051; doi:10.1371/journal.pone.0038967)
Supplement: Figure S1 — Prediction of conformation for Ile234 with the program TALOS+ [35] based on reported chemical shifts. (DOC) [file pone.0038967.s001.doc]

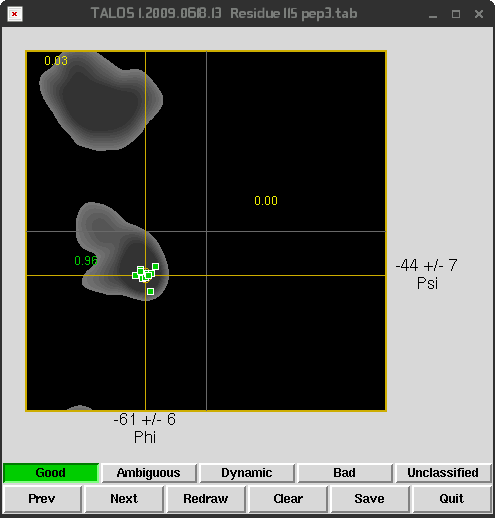


**Figure S1**. Prediction of conformation for Ile15 with the program TALOS+ [35] based on reported chemical shifts.
