# Supplementary material for: Structural Analysis of a Peptide Fragment of Transmembrane Transporter Protein Bilitranslocase
Source: PLoS One. 2012 Jun 20;7(6):e38967. doi: 10.1371/journal.pone.0038967 (PMC3380051; doi:10.1371/journal.pone.0038967)
Supplement: Figure S2 — Distance between Cα atoms in Ala225 and Thr237 calculated on base last 200 ps of trajectory in molecular dynamic simulations performed with the program AMBER 11. (DOC) [file pone.0038967.s002.doc]

**
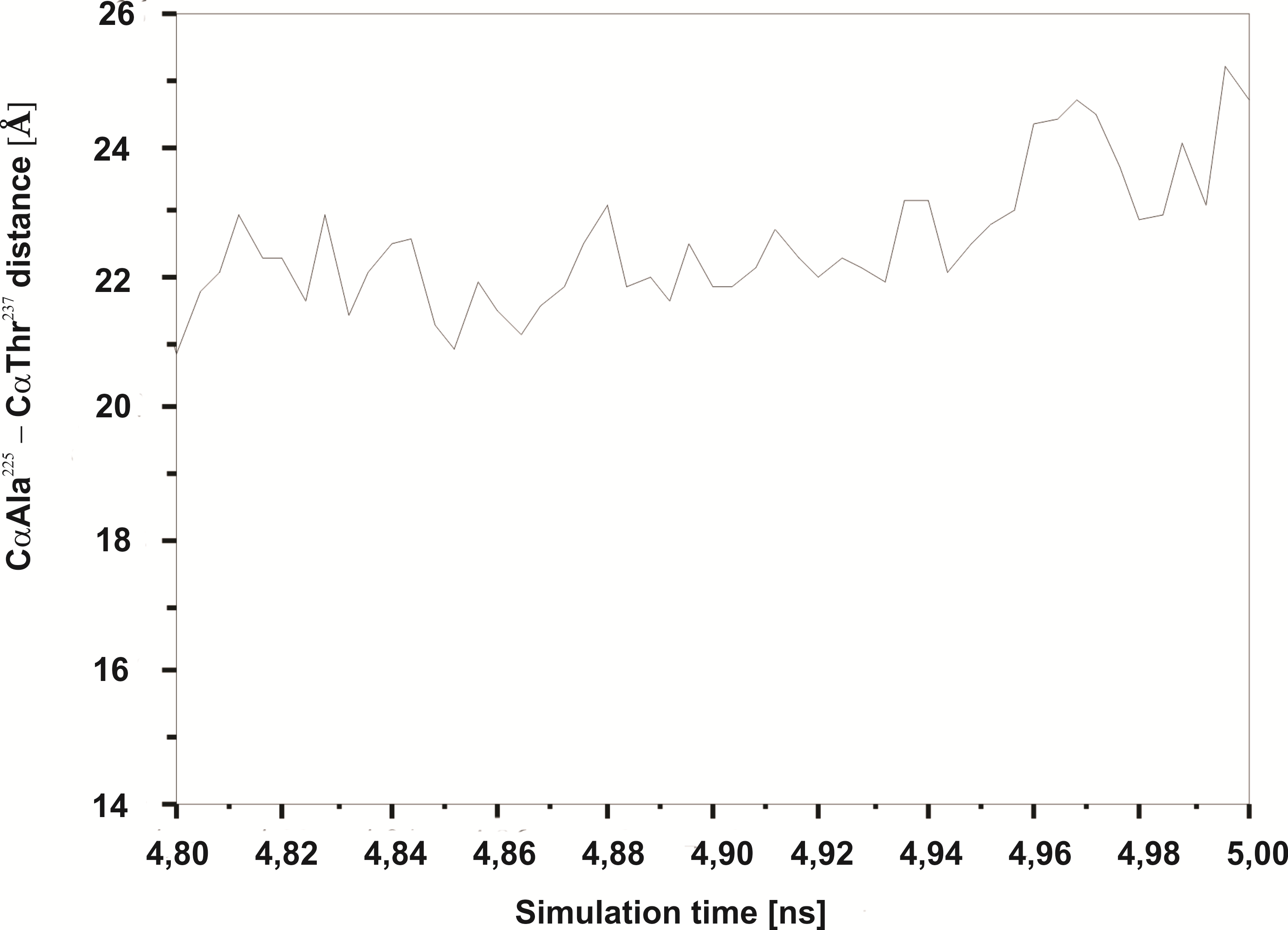
**

**Figure S2.** Distance between C^α^ atoms in Ala225 and Thr237 calculated on base last 200 ps of trajectory in molecular dynamic simulations performed with the program AMBER 11.
