# Supplementary material for: Structural Analysis of a Peptide Fragment of Transmembrane Transporter Protein Bilitranslocase
Source: PLoS One. 2012 Jun 20;7(6):e38967. doi: 10.1371/journal.pone.0038967 (PMC3380051; doi:10.1371/journal.pone.0038967)
Supplement: Table S1 — Scoring all the possible combinations of terminal residues for the third transmembrane stretch of BTL. (DOC) [file pone.0038967.s003.doc]

**Table S1**. Scoring all the possible combinations of terminal residues for the third transmembrane stretch of BTL.

| **Possible TM segments** | **Scores** |
| --- | --- |
| 218 – 230 | -1 |
| 218 – 231 | -3 |
| 218 – 232 | -2 |
| 218 – 233 | -5 |
| 218 – 234 | 0 |
| 218 – 235 | -2 |
| 218 – 236 | -2 |
| 218 – 237 | -4 |
| 218 – 238 | -3 |
| 218 – 239 | -1 |
| 218 – 240 | -3 |
| 219 – 230 | 1 |
| 219 – 231 | -1 |
| 219 – 232 | 0 |
| 219 – 233 | -3 |
| 219 – 234 | 2 |
| 219 – 235 | 0 |
| 219 – 236 | 0 |
| 219 – 237 | -2 |
| 219 – 238 | -1 |
| 219 – 239 | -8 |
| 219 – 240 | -1 |
| 220 – 230 | 3 |
| 220 – 231 | 3 |
| 220 – 232 | 4 |
| 220 – 233 | 5 |
| 220 – 234 | 6 |
| 220 – 235 | 7 |
| 220 – 236 | 9 |
| 220 – 237 | 11 |
| **220 – 238** | **13** |
| 220 – 239 | 6 |
| 220 – 240 | 3 |
| 221 – 230 | 1 |
| 221 – 231 | -1 |
| 221 – 232 | 0 |
| 221 – 233 | -3 |
| 221 – 234 | 2 |
| 221 – 235 | 0 |
| 221 – 236 | 0 |
| 221 – 237 | -2 |
| 221 – 238 | -1 |
| 221 – 239 | -8 |
| 221 – 240 | -1 |
| 222 – 231 | 0 |
| 222 – 232 | 1 |
| 222 – 233 | -2 |
| 222 – 234 | 3 |
| 222 – 235 | 1 |
| 222 – 236 | 1 |
| 222 – 237 | -1 |
| 222 – 238 | 0 |
| 222 – 239 | -7 |
| 222 – 240 | 0 |
| 223 – 232 | 4 |
| 223 – 233 | 1 |
| 223 – 234 | 6 |
| 223 – 235 | 4 |
| 223 – 236 | 4 |
| 223 – 237 | 2 |
| 223 – 238 | 3 |
| 223 – 239 | -4 |
| 223 – 240 | 3 |
| 224 – 233 | -1 |
| 224 – 234 | 4 |
| 224 – 235 | 2 |
| 224 – 236 | 2 |
| 224 – 237 | 0 |
| 224 – 238 | 1 |
| 224 – 239 | -6 |
| 224 – 240 | 1 |
| 225 – 234 | 11 |
| 225 – 235 | 9 |
| 225 – 236 | 9 |
| 225 – 237 | 7 |
| 225 – 238 | 8 |
| 225 – 239 | 1 |
| 225 – 240 | 8 |
| 226 – 235 | 0 |
| 226 – 236 | 0 |
| 226 – 237 | -2 |
| 226 – 238 | -1 |
| 226 – 239 | -8 |
| 226 – 240 | -1 |
