# Supplementary material for: Structural Analysis of a Peptide Fragment of Transmembrane Transporter Protein Bilitranslocase
Source: PLoS One. 2012 Jun 20;7(6):e38967. doi: 10.1371/journal.pone.0038967 (PMC3380051; doi:10.1371/journal.pone.0038967)
Supplement: Table S2 — Structural statistics of distance constraints used for high-resolution 3D structure calculations and quality ensemble of 10 NMR derived structures of 22 residues long peptide on the last steps of MD simulations with TAV. (DOC) [file pone.0038967.s004.doc]

**Table S2.** Structural statistics of distance constraints used for high-resolution 3D structure calculations and quality ensemble of 10 NMR derived structures of 22 residues long peptide on the last steps of MD simulations with TAV.

| Total number of *NOE* restraints | 180 |
| --- | --- |
| Intraresidual (|*i-j*| = 0) | 107 |
| Sequential (|*i-j*| = 1) | 58 |
| Medium-range (|*i-j*| ≤ 5) | 15 |
| Long-range (|*i-j*| > 5) | 0 |
| **Ramachandran plot summary for residues 6 … 18 (%)** | |
| Most favored | 84.5 |
| Additionally allowed | 15.5 |
| Generously allowed | 0.0 |
| Disallowed | 0.0 |
| **Rmsd to the mean structure for residues 6 … 18 (Å)** | |
| Backbone atoms | 0.39 ± 0.15 |
| All heavy atoms | 0.53 ± 0.22 |
