# Supplementary material for: Structural Analysis of a Peptide Fragment of Transmembrane Transporter Protein Bilitranslocase
Source: PLoS One. 2012 Jun 20;7(6):e38967. doi: 10.1371/journal.pone.0038967 (PMC3380051; doi:10.1371/journal.pone.0038967)
Supplement: Text S1 — Prediction of BTL transmembrane regions. (DOC) [file pone.0038967.s005.doc]

Prediction of BTL transmembrane regions

The transmembrane region prediction models predicts for each of the overlapping segments if they are transmembrane or not. However, as the segments are overlapping such that two consecutive ones have 19 amino acids in common, a transmembrane region is only predicted if 10 or more consecutive overlapping segments are reported as transmembrane. Individual or short stretches of overlapping segments predicted as transmembrane are ignored because they overlap with other segments predicted as non-transmembrane. Similarly, isolated predictions of non-transmembrane segments within long stretch of transmembrane regions are also not considered.

As the segments are overlapping, not all the residues in the stretch of overlapping segments are predicted to be in the final transmembrane region. Only residues that are common to more segments predicted as transmembrane rather than non-transmembrane are ultimately considered to form the transmembrane region, while the preceding and trailing residues are disregarded. Therefore, only the central residues of the predicted transmembrane stretches are reported as the final transmembrane regions.

The problem with this initial prediction is that the transmembrane regions are assumed to contain only the central residues of the long stretch of overlapping segments predicted as transmembrane. Therefore, to obtain transmembrane region terminals that are more statistically favored and closer to experimental data, we consider all possible combinations of terminal residues of the segments predicted as transmembrane. These terminal combinations are then scored and the best scoring and positioned region is selected as final prediction.
