# Supplementary material for: Structural Analysis of a Peptide Fragment of Transmembrane Transporter Protein Bilitranslocase
Source: PLoS One. 2012 Jun 20;7(6):e38967. doi: 10.1371/journal.pone.0038967 (PMC3380051; doi:10.1371/journal.pone.0038967)
Supplement: Text S2 — Statistical calculation of amino acid position preference. (DOC) [file pone.0038967.s006.doc]

Statistical calculation of amino acid position preference

Our dataset consists of transmembrane regions for 101 alpha transmembrane proteins. A position specific amino acid preferrence calculation was done for 5 positions each at the N- and C-terminals of the transmembrane regions. This generates a list of amino acids preferred or avoided in each of these 10 terminal positions. This is then compared with a similar statistical calculation done on alpha helices of globular proteins. The amino acids at a position that exclusively prefer or avoid the particular position when compared with other positions of transmembrane regions and same position in globular alpha helices are designated as critically preferred or avoided. A positive score of +1 is given if a preferred amino acid is present at a particular residue position of the terminal, and negative score of -1 is given if it contains a statistically avoided amino acid. Similarly, scores of +2 or -2 are given if the amino acids present are critically preferred or avoided, respectively.
