# Supplementary material for: Structural Analysis of a Peptide Fragment of Transmembrane Transporter Protein Bilitranslocase
Source: PLoS One. 2012 Jun 20;7(6):e38967. doi: 10.1371/journal.pone.0038967 (PMC3380051; doi:10.1371/journal.pone.0038967)
Supplement: Text S3 — Comparison with other predictors. (DOC) [file pone.0038967.s007.doc]

Comparison with other predictors

The transmembrane region prediction of BTL by our model was compared with the results from other available predictors [23]. The results showed that only 3 predictors (TMpred, TopPred, and TMpro) other than the developed one were able to predict four proposed transmembrane regions of BTL. Most other predictors predicted BTL to have 2 (SVMtm, Phobius, ConPred, igTM, HMMTM), 3 (MEMSAT, HMMTOP, Ohilius, CoPreTHi, ), or 5 (PHD) transmembrane regions. TOPCONS predicted 3 transmembrane regions and failed to predict TM 2 of BTL. TMHMM on other hand was unable to predict both the second and third transmembrane regions of BTL. Interestingly, all predictors were able to predict TM 1 and TM 4, while having problems in predicting TM 2 and TM 3, the transmembrane regions that are proposed to form the transport channel [6,22,24].
