# Supplementary material for: Structural Analysis of a Peptide Fragment of Transmembrane Transporter Protein Bilitranslocase
Source: PLoS One. 2012 Jun 20;7(6):e38967. doi: 10.1371/journal.pone.0038967 (PMC3380051; doi:10.1371/journal.pone.0038967)
Supplement: Text S4 — Details on Counter-propagation neural network model. (DOC) [file pone.0038967.s008.doc]

Details on Counter-propagation neural network model

The model uses a dataset of 5800 protein segments (2545 transmembrane and 3255 non-transmembrane), of which 4204 segments were used to train it. The remaining 450 and 1146 segments were used for internal and external validations, respectively. Each protein segment is encoded in a 20×20 amino acid adjacency matrix. The rows and columns are labeled by one letter code of 20 amino acids and the non-zero elements represent the subsequent pairs in the sequence of the polypeptide. The appearance of each pair of amino acids enlarges the matrix element at the position of these particular amino acids by one. The 20-element row-sum vector derived from the matrix acts as the 20 mathematical descriptors defining the particular segment. A 40×40 network with maximum correction factor 0.9 and trained for 500 epochs was chosen to be optimum. The model predicts the segments as transmembrane or non-transmembrane with 90.75% accuracy for the external validation set [22].
